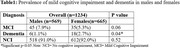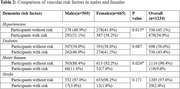# Sex differences in the prevalence of mild cognitive impairment and dementia, and their risk factors, in Bengaluru

**DOI:** 10.1002/alz.091143

**Published:** 2025-01-09

**Authors:** Aparna Lalana Venugopal

**Affiliations:** ^1^ National Institute of Mental Health and Neurosciences, Bengaluru, Karnataka India

## Abstract

**Background:**

The burden of dementia is expected to increase owing to the rapidly increasing number of aged populations across the world. It is widely reported that risk of developing dementia is high in women compared to men and the contributing factors resulting these discrepancies are not well understood. In this context, the goal of the current study was to assess whether the prevalence of mild cognitive impairment (MCI) and dementia differs by sex in urban Bengaluru and whether risk factors for MCI and dementia differ by sex.

**Method:**

A community‐based, cross‐sectional study was conducted in urban Bengaluru between January 2021 and December 2021. The study team performed household enumeration to identify individuals who were **≥** 60 years old. Neurologic, neuropsychological, and functionality tests and a risk profile questionnaire were administered to all participants. The assessment tools included Addenbrooke’s Cognitive Examination (ACE‐III; Mekala et al., 2020; Mathuranath et al., 2007), Clinical Dementia Rating Scale (CDR; Morris, 1991), and Instrumental Activities of Daily Living‐Elderly (IADL‐E; Mathuranath et al., 2005). The risk profile questionnaire included screening for common cardiovascular risk factors such as hypertension, heart disease, diabetes and stroke. Participants were categorized as having no cognitive impairment (NCI), MCI, or dementia. MCI was diagnosed based on Petersen’s criteria (2004) and dementia was diagnosed based on DSM‐IV criteria (1994).

**Result:**

Of the 1,234 participants, 665 (53.9%) were female and 569 were male (46.1%). Males were slightly older (mean (SD): 72.0 (8.68) vs. 70.5 (7.79) years, p = 0.001) and had more years of education (mean (SD): 13.3 (4.65) vs. 9.9 (5.75), p<0.001). Of the 1,234 participants, 80 had MCI (6.5%) and 24 (1.9%) had dementia. More males than females had MCI (7.9% vs. 5.3%, p = 0.06). In contrast, males were less likely to be diagnosed with dementia (1.1% vs. 2.7%, p = 0.04). Males had a higher prevalence of heart disease, whereas females had a higher prevalence of hypertension.

**Conclusion:**

The prevalence of MCI and dementia differs by sex in Bengaluru, as does the prevalence of vascular risk factors.